# Current Overview on the Use of Mesenchymal Stem Cells for Perianal Fistula Treatment in Patients with Crohn’s Disease

**DOI:** 10.3390/life11111133

**Published:** 2021-10-25

**Authors:** Marcin Włodarczyk, Katarzyna Czerwińska, Jakub Włodarczyk, Jakub Fichna, Adam Dziki, Łukasz Dziki

**Affiliations:** 1 Department of General and Oncological Surgery, Medical University of Lodz, Pomorska St. 251, 92-213 Lodz, Poland; katarzyna.czerwinska@stud.umed.lodz.pl (K.C.); jakub.wlodarczyk@stud.umed.lodz.pl (J.W.); lukasz.dziki@umed.lodz.pl (Ł.D.); 2 Department of Biochemistry, Medical University of Lodz, Mazowiecka 6/8, 92-215 Lodz, Poland; jakub.fichna@umed.lodz.pl; 3 Department of General and Colorectal Surgery, Medical University of Lodz, Zeromskiego St. 113, 90-057 Lodz, Poland; adam.dziki@umed.lodz.pl

**Keywords:** mesenchymal stem cells, perianal fistula, Crohn’s disease, perianal Crohn’s disease

## Abstract

Perianal fistula in patients with Crohn’s disease is an extremely challenging condition. The disease tends to reoccur, and with current treatment options, a large number of patients are left with active ailment and experience major morbidity. In recent years, hopeful results regarding local use of mesenchymal stem cells (MSCs) in perianal Crohn’s disease have been published. Although to this day there are no clear guidelines determining optimal dosage, injections frequency and culture conditions, their efficiency has proven to be much higher than conventionally used methods. According to studies, they can effectively induce as well as maintain fistula closure. This approach also avoids common side effects related to conventional surgical treatment.

## 1. Introduction

Crohn’s disease (CD) is a relapsing systemic inflammatory disease that can cause persistent transmural inflammation anywhere along the gastrointestinal tract. A substantial number of CD patients present with various anorectal pathologies including perianal abscesses, fistulas, hemorrhoids, skin tags and fissures [1]. Perianal fistulas are described as an abnormal connection between the anal canal and the perianal skin.

According to population-based studies, around one in every four CD patients will develop perianal fistula at some point in their life. Patients with colonic or rectal localization of intestinal inflammatory lesions are at an even higher risk, at about 92% [2]. Diagnosis and management of perianal Crohn’s Disease (PCD) requires an expert, multidisciplinary approach, considering that most of the perianal fistulas are defined as complex (meaning they involve the upper part of the sphincters, are complicated with a perianal abscess, or have multiple external openings or penetrate to vagina). These patients’ quality of life is considerably impaired due to persistent drainage, pain, recurrent perianal sepsis and continuous need to seek medical attention [3]. Patients with perianal fistula are also more likely to have extraintestinal manifestations of CD including arthritis, oral ulcerations and skin manifestations.

The management of PCD remains a challenge, as 37% of patients experience refractory disease. The relapse rate is estimated at around 40% in 5-year follow-ups [4]. For this reason, many patients are exposed to the necessity to repeatedly take immunosuppressive medications, which increases the risk of opportunistic infections. Furthermore, about 90% of them undergo numerous surgical interventions, becoming liable to complications, including fecal incontinence [2,4]. Even with combined pharmacological and surgical therapy, 40% of patients do not achieve remission and are left with active disease, facing the risk of undergoing proctectomy.

This perspective encouraged us to seek more desirable treatment options to provide higher therapy effectiveness with a lack of adverse effects and lower risk of incontinence. Recently, promising results using local injections with mesenchymal stem cells (MSCs) have been reported. This treatment has been proven to effectively induce as well as maintain fistula closure [5,6].

MSCs are stromal cells that have the ability to self-renew and differentiate into adipocytes, myocytes, osteocytes and chondrocytes. Moreover, they have powerful immunomodulatory effects and are able to reduce escalated inflammation, as they inhibit the proliferation and function of T, B and NK cells [6]. MSCs are present in almost all tissues; however, they are most commonly isolated from bone marrow, adipose tissue or an umbilical cord. In the last decade, physicians have been able to activate and supplement these cells to treat a variety of conditions, for instance, many autoimmune diseases. At present, multiple new studies on the use of MSCs for Multiple Sclerosis, chronic obstructive pulmonary disease, cancer, lupus and Parkinson’s are being carried out [6].

The aim of this systematic review is to evaluate the available results from clinical studies, regarding MSCs use in PCD, point out the advantages and disadvantages of this method and also indicate the necessity for further research.

## 2. Methods

### 2.1. Searching Strategy

**Figure 1:**
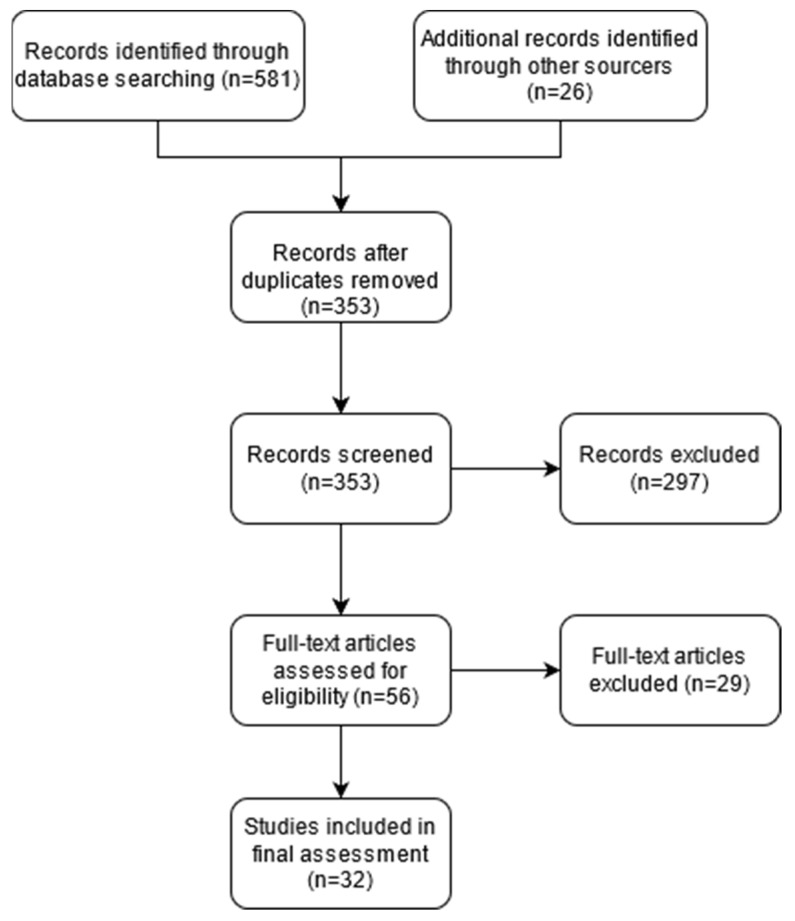
Workflow of identification of studies relevant to the mesenchymal cell use in perianal Crohn’s Disease.

In a systematic review of PubMed, search terms were selected to identify literature on MSCs use in PCD. Results were limited to relevant papers published in English. A total of 581 studies were retrieved, plus 26 studies derived from review articles. There were no restrictions on the publication date for the articles cited in all subsections of the manuscript. The first search was performed on 1 November 2020, and the search was updated on 30 December 2020, with a final revision on 20 February 2021. Figure 1 delineates the workflow from the initial searches to the 32 studies ultimately included in the analysis, in accordance with PRISMA guidelines [7].

### 2.2. Study Selection and Risk of Bias

The references in all the included studies were reviewed for more eligible articles. Each article was reviewed independently by three researchers (M.W., K.C., J.W.) for inclusion according to the inclusion and exclusion criteria, which follow. Disagreements regarding article selection were resolved through discussion until consensus was reached or resolved by discussion between authors M.W., A.D. and L.D. Prospective and retrospective observational human studies on adult patients were included. Conference abstracts were excluded. Articles were also excluded if they were not in English, the full text was inaccessible or the studies were preclinical research or commentaries. A standardized form was used to extract data from the included studies. Extracted details were study population and demographics, details of interventions and controls, study methodology and information to assess bias. Data extraction was performed independently by four authors, and discrepancies were resolved through discussion with the other co-authors.

### 2.3. Outcome Assessment

The main outcome was the evaluation of available clinical studies results, regarding MSCs’ use in PCD, pointing out advantages and disadvantages of this method and also indicating the necessity for further research.

### 2.4. Ethical Considerations

Ethics committee approval was not required for this study because it was a systematic review. Patient consent was not required because no patients or patient-identifiable data were involved in the study.

## 3. Mesenchymal Stem Cells Mechanism in Crohn’s Disease Perianal Fistula

To this day, the exact mechanism in which MSCs heal perianal fistulas remains unknown, as no human studies have clarified it. It likely results from their immunomodulatory properties [8]. Firstly, they migrate to the sites of injury or inflammation and directly spur tissue healing by tissue specific differentiation and the secretion of factors with the ability to promote epithelial cell proliferation and angiogenesis. MSCs help maintain an anti-inflammatory habitat by modulating the function of macrophages, lymphocytes and dendritic cells.

Regarding CD, MSCs’ ability to upregulate the CD4+ T cell subset is particularly important, as patients with CD are known to have a deficiency of those cells [9,10]. Furthermore, MSCs secrete various anti-inflammatory particles (including TGFB1, growth factors, interleukins and indoleamine 2,3-dioxygenase) and suppress M1 macrophages, cytotoxic T cells and dendritic cells [11]. They play a significant role in healing of the fistula, owing to their role in angio- and mitogenesis, as well as immunomodulatory effect [12]. They reduce inflammation in tissues around the fistula and accelerate healing. Due to its’ promising preclinical studies results, clinicians are more and more interested in CD treatment using MSCs.

## 4. Mesenchymal Stem Cells Application in Perianal Crohn’s Disease

Regrettably, there is no clear surgical protocol for the administration of stem cells in PCD therapy. To start with, there are no studies comparing side by side the use of adipose- vs. bone-marrow-derived MSCs. Adipose-derived stem cells are usually preferred because they are much easier to harvest with the use of liposuction. Furthermore, they are known to have a higher replication rate and longer proliferation in culture [5,13]. They might also induce a stronger immunomodulatory effect, due to the fact they have higher secretion levels of cytokines, including TGFB1 and IL-6 [9].

MSCs are typically used locally, to avoid side effects and help keep cells in direct contact with inflamed tissue [14]. In that case, either autologous or allogenic cells can be used. During treatment with the use of autologous cells, extraction and application happens in the course of the same procedure [13,15]. Thus, MSCs are injected without previous expansion, which results in their low dose [16].

According to a meta-analysis based on four clinical trials involving different doses of stem cell therapy ranging from 1 × 10^7^ to 9 × 10^7^ MSCs per mL, the dose of 2 × 10^7^ and 3 × 10^7^ cells/mL were characterized by a highest rate of fistula healing in Crohn’s patients [17]. However, the mentioned study is strongly limited by a low number of included patients (*n* = 31). Due to the lack of other studies based on a larger group, it is difficult to propose specific guidelines regarding the dosage of MSCs. Therefore, it is crucial to continue such studies.

However, allogenic cells are endowed with excellent homogeneity [18]. They might not create antibodies, which would be clinically relevant. Cells are previously expanded and frozen. It only takes 4 days to defrost them and prepare for administration. Allogenic MSCs are also considered particularly beneficial in CD.

To date, no definite conclusion about an optimal dosage has been made. According to Molendijk et al.’s study, the best results are achieved with the use of 30 million cells [19]. The most commonly used concentration contains 5 × 10^6^ MSCs per mL; however, no studies have directly compared the outcome determined by the use of different MSCs concentrations.

Preoperatively, each patient should be thoroughly examined, to prepare a precise description of the fistula tract. It is necessary to estimate the needed MSCs volume. A magnetic resonance imaging or endorectal ultrasonography (ERUS) can be found helpful to provide information concerning the type, location and presence of branches of the fistula.

Prior to surgery, any active infection should be controlled by antibiotics, and any abscesses greater than 2 cm should be incised and drained. Fistula biopsy should also be considered to rule out any type of local neoplasia, since MSCs might promote tumor growth. There is no reason to discontinue systemic treatment for the CD.

As far as anesthesia is concerned, most frequently used local anesthetics such as lidocaine can cause a cytotoxic effect to the MSCs when in direct contact. For that reason, local anesthesia ought to be avoided. If necessary, the recommended approach is to perform a pudendal block. This concern should also be kept in mind if the MSCs are obtained by liposuction. During this procedure, the use of local anesthetics is discouraged, as they weaken the MSCs’ immunomodulatory properties and viability.

The antiseptic used to prepare the surgical site must not be harmful to the cells. Thus, the use of alcoholic, povidone solutions or hydrogen peroxide is discouraged. Rather, it is advised to use octenidine, chlorhexidine or normal saline.

The next step is to identify the internal fistula opening using a probe, or by injecting normal saline through the external orifice. To create an optimal environment for healing, the fistula tract needs to be mechanically debrided using curette and irrigated with saline. Afterwards, the internal opening needs to be securely closed with the use of an absorbable suture.

**Figure 2:**
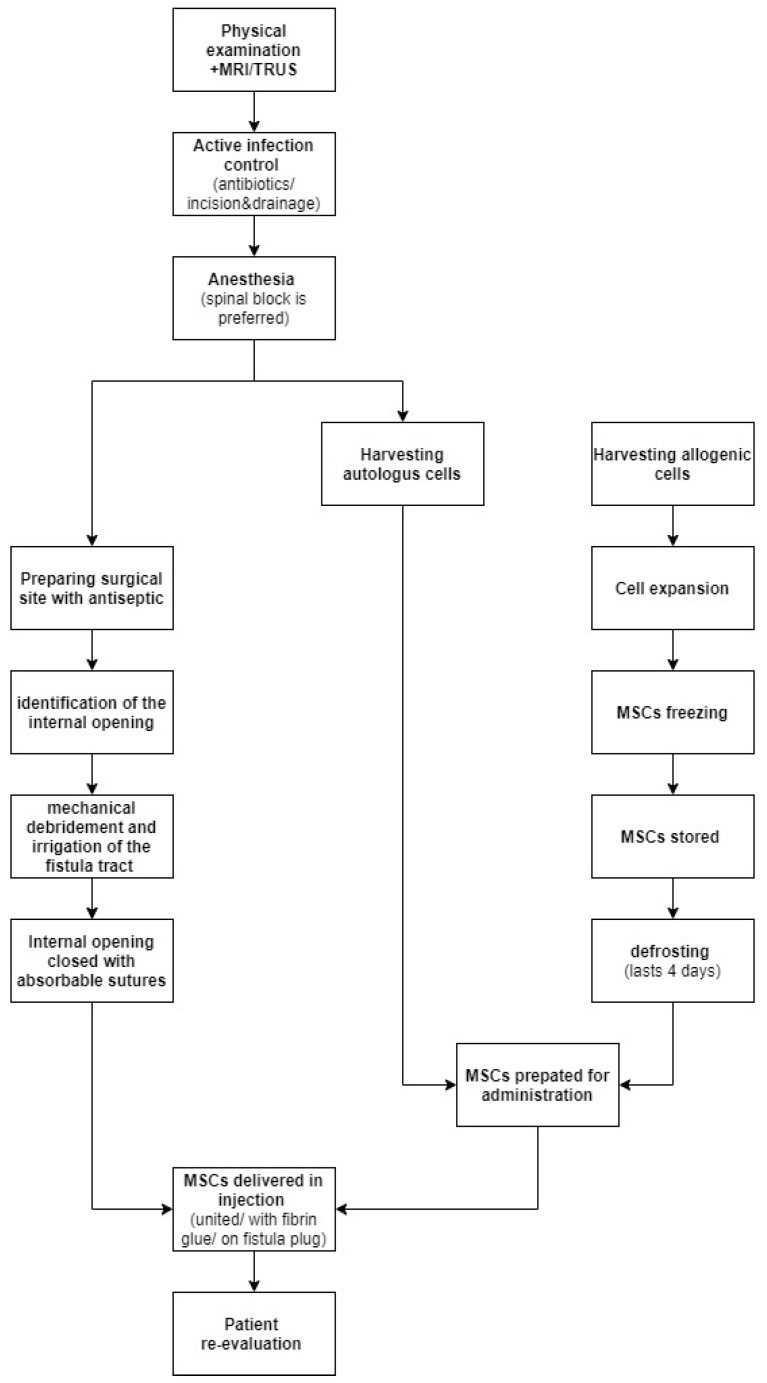
MSCs application process.

MSCs can be delivered in direct injections, united with fibrin glue or impregnated on a fistula plug. It is believed that healing rates are higher when scaffolding material is used, due to the fact that glue or plug help maintain MSCs at the desirable location. On the other hand, a randomized clinical trial involving 200 patients comparing the efficiency of treatments using MSCs, MScs mixed with fibrin glue or fibrin glue alone proved there are no significant differences in the healing rate with the use of these three methods. In Figure 2 we have provided a general overview regarding MCSc application process.

## 5. Analysis of Recent Clinical Studies Outcomes

### 5.1. ADMIRE-CD

Several studies evaluated the efficiency of MSCs in perianal fistula treatment in patients with Crohn’s disease. Most were single-armed and involved a low number of patients. To date, the largest published trial is entitled ADMIRE- CD [20,21] (Adipose-Derived Mesenchymal Stem Cells for Induction of Remission in Perianal Fistulizing Crohn’s Disease). It is a randomized, double blind, placebo-controlled study, which involved 212 adult patients. Each participant suffered from luminal CD and had a complex perianal fistula, which did not respond to previous conventional treatments.

Around 2 weeks before MSCs treatment, every patient underwent fistula preparation including curettage and seton placement if needed. During the essential surgical procedure, firstly the internal opening was closed with absorbable stitches, and then one group (consisting of 107 patients) received injections with 120 million Cx601 cells along the fistula tracts. The placebo group (105 patients) was injected with 24 mL of saline solution.

Primarily, the patients were observed for 24 weeks. The endpoint was established as the closure of all the external openings and the absence of abscesses larger than 2 cm in diameter, verified by an MRI. The endpoint was notably more frequently achieved by patients in the Cx601 group (50% vs. 34% in the control group). Furthermore, remission occurred in a shorter amount of time (Median 6.7 weeks in the Cx601 group and 14.6 weeks in the placebo group). As far as treatment-emergent adverse effects are concerned, the most common were proctalgia, anal abscess and nasopharyngitis. They occurred in 17% of patients in the Cx601 group and 29% in the placebo group. Serious TEAEs (Treatment-Emergent Adverse Events) were experienced, respectively, by 17 and 14% of patients. It is worth noting that there were no adverse effects related directly to the stem cells themselves.

Effectiveness from week 24 was confirmed in the annual follow-up. Combined radiological and clinical remission occurred in 56% of the patients that were treated with stem cells, and 39% in the patients that received saline injections. Moreover, the relapse rate by week 52 in the patients that obtained remission at week 24 was 46% higher in those treated with stem cells. TEAEs were noted in similar percentage in both groups (77 and 73%), but serious TEAEs were slightly more common in the Cx601 group (24% vs. 21% in the control group).

The ADMIRE-CD study led to the approval of the first commercial solution of MSCs to be used in PCD (Alofisel, Takeda, Zurich, Switzerland) by the European Medicine Agency (EMA). The European commercially available solution is constituted of four vials of 6 mL of MSCs with 30 million cells each, with a total of 120 million cells [22]. The complexity of the procedures is linked to the short shelf life of cell viability, approximately up to 72 h from the laboratory facility to the operating room, with a significant rise in the cost of the MSCs.

### 5.2. 2009. Garcia-Olmo 

A phase II, randomized study, including 14 patients with complex perianal fistulas and Crohn’s disease [23]. They were randomly assigned to local treatment with fibrin glue alone or with addition of 2 × 10^7^ autologous stem cells harvested from adipose tissue. Fistula healing was evaluated at week eight and after one year. At the 8-week visit, five of the seven patients (71%) that were treated with MSCs achieved full closure of the fistula tract. Out of the seven patients in the control group, only one (14%) was successfully healed.

If complete epithelialization of the external opening was not seen at eight weeks, the patients received a second injection with fibrin glue or fibrin glue with a double dose of MSCs. At the 12-month follow-up, the recurrence rate in the patients treated with MSCs was 17.6%.

### 5.3. 2013. Lee, Park and Cho Study and 2015 Cho, Park, Yoon Study

An open-label, phase II study, which included patients diagnosed with perianal fistulae associated with Crohn’s disease [24,25]. The participants were injected with MSCs into the lesion site. Autologous stem cells were obtained from adipose tissue. The patients were monitored for 8 weeks and then attended an additional 10-month follow-up visit. The endpoint was established as full epithelialization of the external opening with no signs of inflammation or drainage.

The dose of MSCs was 3 × 10^7^ or 6 × 10^7^ depending on the fistula size. If the fistula was not completely closed at week eight, a second injection was performed, using a 50% higher number of cells.

At week eight, 79% (26/33) of the patients showed complete fistula healing. A second injection was given to the remaining seven patients, and one of them achieved fistula closure after another eight weeks. Out of six patients that did not show complete closure of fistula, five of them had healed more than 50% of the fistula tract and stated a significant decrease in drainage. A total of 26 patients completed a 12-month study. At a 1-year follow-up visit, fistula healing was sustained in 23 of them (88.5%).

This study was extended by Lee, Park and Cho. In 2015, they published paper demonstrating the long-term results of this MSCs treatment. Patients were observed for another year. Out of the 24 participants that completed this follow-up, 20 (83.3%) maintained a complete closure.

### 5.4. 2013. De la Portilla et al. Study

It is an open-label, single-arm clinical trial [26]. A total of 24 patients were administered with 2 × 10^7^ expanded allogeneic adipose-derived stem cells in one draining fistula tract. If the fistula was not completely closed at week 12, the participants were injected with additional 4 × 10^7^ ASCs. This approach was required in 15 patients. Subjects were followed for 6 months after the first stem cell application.

A reduction in the number of draining fistulas was presented by 60% of the patients in week 12, and 69% in week 24. During the 12-week follow-up visit, 38.1% of the participants showed complete closure of the external opening of the treated fistula. Data corresponding to week 24 were 56.3%, with no significant difference between the patients that received one or two doses of MSCs. Full fistula closure (defined as the absence of suppuration, complete re-epithelization, and no fluid collections >2 cm visible in an MRI scan) was achieved by 30% of the patients at the 24^th^ week. No subject presented luminal relapse during the first 12 weeks of the trial; however, five patients (20.8%) showed relapse by the sixth month.

### 5.5. 2017. Dietz et al.

It was a 12-patient, 6-month, phase 1 trial [27]. The aim of this study was to determine if autologous MSCs, applied in a bioabsorbable matrix, are able to heal perianal fistula. Eligible patients suffered from persistent refractory Crohn’s Disease with presence of a single draining fistula for at least 3 months. Patients with proctitis were excluded. Autologous cells were obtained from adipose tissue. After 6 weeks, each patient had an MSCs loaded plug placed into the fistula. Every matrix contained around 2 × 10^7^ MSCs.

After 12 weeks, nine of the twelve patients (75%) achieved complete healing, defined as full discontinue of drainage. At a 6-month visit, 10 of 12 patients (83%) had radiographic response and fistula healing. Radiographic response refers to a decrease in both the diameter and length of the fistula tract. Moreover, the patient must not have developed an abscess or presented an increase in the Van Assche MRI score. In the responding patients, the mean decrease in fistula length was 23.5 mm, and the diameter was averagely reduced by 5 mm.

### 5.6. 2015. Molendijk et al.

This double-blind, placebo-controlled trial evaluated effects after a local administration of MSCs to patients with actively draining perianal fistula associated to Crohn’s disease [19]. Mesenchymal cells were obtained from bone marrow aspirates of healthy donors. During the study, 21 patients were randomly assigned to four groups, and administered a different number of MSCs.

Before the essential procedure, each patient had a temporary seton placed and any present abscesses > 2 cm were drained. Main surgery was performed under general anesthesia. MSCs injections were preceded by identification of the internal opening, seton removal, debridement of the fistulous tracts and closure of the internal opening with an absorbable suture. Afterwards, half of the solution (with content appropriate for each group) was injected around the internal opening, and the other half into the anal wall, close to the fistula tract.

The patients were than evaluated after 6, 12 and 24 weeks. At every appointment, the fistula was checked for discharge, and at week 12, the participants underwent an MRI to reveal any fluid collection. Primary outcome—fistula healing was defined as the absence of discharge and no fluid collections > 2 cm. The results are presented in a Table 1.

During the study period, all the adverse events were recorded. All the patients described pain and discharge in the first week after surgery. Four patients (each from every group) developed perianal abscesses that needed to be drained. Three patients from the placebo group complained of a painful perianal swelling and had to be treated with antibiotics. None of these reported adverse effects were found to be related to the MSC injections.

In summary, local treatment with 3 × 10^7^ MSCs (group two) showed greater fistula healing compared with the placebo, and lower doses of MSCs seemed to bring superior results in comparison with high doses.

### 5.7. 2020. Barnhoorn et al.

This study provided long term follow-up for thirteen patients that participated in the 2015 Molendijk, Bonsing, Roelofs study [28]. They belonged to cohorts one, two and three and received injections containing MSCs. No one from the placebo group was involved in this study.

The patients were observed for an additional 3.5 years. They underwent a proctoscopy and pelvic MRI. The results showed that the treatment efficiency reported at week 24 was maintained after 4 years in groups two and three. In group one, statistics have improved, and all the patients managed to have full fistula closure. According to CDAI (Crohn’s Disease Activity Index), disease activity was lower 4 years after MSCs therapy. Additionally, each patient’s quality of life was assessed using Short Inflammatory Bowel Disease Questionnaire and compared with results from before the treatment. The results showed significant improvement in the quality of life. As far as adverse events are concerned, four patients developed perianal abscess and five were treated for infections. CD activity occurred in three participants.

### 5.8. 2020. Laureti et al.

This prospective research involved 15 patients who suffered from complex, refractory PCD [29]. During this study, each of them was injected with autologous micro-fragmented adipose tissue. They were subsequently assessed 2, 4, 8, 12 and 24 weeks after treatment. The primary end point was defined as the absence of fluid collections >3 mm confirmed in MRI scans and the closure of all the treated external fistula openings at week 24. This combined remission was achieved by 10 (66.7%) patients. A lack of draining fistula alone appeared in 14 (93.3%) patients. These results were maintained at a 24-months follow-up visit. No major complications were noted. A total of 20% of the participants experienced subcutaneous hematoma related to lipoaspiration, which resolved spontaneously.

### 5.9. 2020. Zhou et al.

A total of 22 patients with complex perianal fistulas related to CD were enrolled in this study [30]. They were divided into two equal groups, one receiving autologous, adipose-derived stem cells, and the other underwent an incision-thread-drawing procedure. During the study, all the participants were treated with aminosalicylic acid and a probiotic. The patients were assessed 3, 6 and 12 months after the procedure. The assessment contained a clinical examination and MRI or ERUS. The endpoint was defined as no evidence of fistulas in an ERUS/MRI and complete epithelialization of external openings. The results showed no significant difference between the observation and control groups. The healing rate at a 1-year visit after adipose-derived stem cell treatment was 63.6% vs. 54.5% in the control group. Adverse effects occurred in 64% of the patients in the observation group, and all the patients in the control group, most commonly pyrexia, perianal pain and fatigue.

### 5.10. Drawbacks

This new therapy demands a large financial contribution. A surgical procedure can be found relatively easy; however, the preparation for this therapy takes a lot of time and requires specialized laboratories. Donors must be carefully selected, cells harvested and then expanded and stored. Due to the fact MSCs are living cells, they need to be handled with care. If not frozen, they are available only for 72 h and must be stored at a temperature between 15 and 25 °C. They also must not be exposed to direct light. Furthermore, as MSCs cannot be sterilized, they hold a potential risk for containing contaminated biological material. They also have a slight risk for cellular transformation, which can lead to their unpredictable behavior.

Analyzed studies showed that during the procedure, a large number of stem cells are lost [31]. It can significantly reduce the efficiency of procedure. This happens because cells do not have enough access to blood supply. MSCs need to be within ~200 µm of the nearest blood vessel to receive sufficient oxygen and nutrients. Moreover, the preparation’s consistency does not provide enough structural support to the injected cells, which results in their poor retention [32]. In the future, both the retention and survival of MSCs after their local administration must be upgraded to improve the therapeutic outcome.

In summary, even though MSC treatment is associated with many difficulties, it is undeniably one of the most promising treatment options for the treatment of PCD. In Table 2, we gathered the mentioned literature regarding the usage of MSCf for perianal fistula treatment in patients with Crohn’s disease.

### 5.11. Secondary Outcomes

Recent studies have also focused on other related scores and indicators of perianal Crohn’s disease regarding the mesenchymal stem cell therapy. Meta-analysis by Cao et al. and Wange et al. assessed the parameters such as CDAI (Crohn’s Disease Activity Index), PDAI (Perianal Crohn Disease Index), IBDQ (inflammatory bowel disease questionnaire) score and CRP (C-reactive protein). They confirmed that the CDAI scores were significantly lower in the transplantation groups compared to the control groups. However, Cao et al. stated that after the administration of stem cells, a transient rise of the CDAI score appeared. The highest peak was observed after 1 month of therapy, while after 3 months it lowered below the baseline score. The PDAI score was also significantly lower after the implementation of stem cell therapy. The quality of life among Crohn’s disease patients significantly increased after MSC administration; however, the increase was not observed until 6 months and reached a peak after 12 months of therapy. Regarding the CRP levels, Cao et al. suggest a continuous drop of the CRP level after stem cell therapy, while Wang et al. report that levels did not differ before and after the treatment.

## 6. Limitations of the Paper

Several limitations of our review should be acknowledged: (1) In clinical trials, they did not report the results about immunohistochemistry and endoscopy in quantification. (2) The subgroup analysis was inadequate owing to the majority of studies included about cohort studies. (3) Given the limited follow-up among the included studies, we failed to elaborate the recurrent after administrating stem cells among CD patients. (4) Our paper is a literature review without statistical analysis and no quality assessment.

## 7. Conclusions

The studies concluded that MSC therapy is one of the promising new treatment options for CD patients, making MSC therapy an exciting new treatment option for CD patients with CD with complex, treatment-refractory perianal fistulas. One may argue that these findings may be able to transfer over to CD patients, as a reduction in inflammation may decrease symptoms and lower the chance of further flare-ups. MSC therapy is a safe treatment and has a strong immunomodulatory effect, meaning they can prevent the immune system from mistakenly responding to incorrect threats. This can reduce negative immune responses in patients with CD. To date, MSC treatment displays the highest efficiency of all the used methods [33,34]. This minimally invasive approach also avoids obnoxious side effects related to conventional surgical treatment, such as fecal incontinence or severe scarring. The frequency of relapses seems to decrease. Even though there are no studies confirming the long-term efficiency of MSC treatment, histopathological samples obtained from previously affected perianal areas, from patients that underwent MSC therapy, showed the presence of a normal epithelium and smooth muscles with a high number of collagen fibers. There were also no signs of MSCs rejection, which indicates that there is a high chance they will provide a positive long-term outcome [35].

Although MSCs’ efficiency in treating PCD is much higher than other conventionally used methods, they are, up until now, not fully satisfying. There are still many uncertainties concerning the use of MSCs. There are no clear guidelines regarding an optimal dosage or the eventual need to repeat MSCs injections for the finest therapeutic outcome. Moreover, an optimal profile of a suitable patient is yet to be determined.

Creating a multidisciplinary team seems to be the key in treating patients with perianal fistulas related to CD. To achieve the best treatment results, a pharmacological approach and surgical preparation of the fistula tract need to be coordinated and adjusted to the specific method in which MSCs are going to be used.

## Figures and Tables

**Table 1:** Healing rate depending on the number of injected MSCs.

Group	Number of Injected MSCs	Healing Rate (%)		
		Week 6	Week 12	Week 24
1	1 × 10^7^	60	40	80
2	3 × 10^7^	80	80	80
3	9 × 10^7^	20	20	20
placebo	0.9% NaCl/5% human albumin solution consisting no cells.	16.7	33	33

**Table 2:** Reviewed literature summary.

Authors	Year	Patients	Cell Type	Aplication/Intervention	Time-Point	Healing Rate (%)	Follow-Up	Recurrence Rate %
ADMIRE-CD study [20,21]	2016, 2018	212	Adipose allogenic (Cx601)	Local application of 120 million Cx601 cells vs. placebo (control group)	24 weeks	50% vs. 34% in control group	1 year	25% vs. 44.1% in control group
Garcia-Olmo et al. [23]	2009	14	Adipose autologous	Local application of 2 × 10^6^ stem cells+ fibrin glue	8 weeks	71%	52 weeks	17.6%
Lee et al. [24,25]	2013 2015	33	Adipose autologous	Local application of 3 × 10^7^ or 6 × 10^7^ stem cells	8 weeks	81.8%	1 year 2 year	11.5% 16.7%
De la Portilla et al. [26]	2013	24	Adipose allogenic	Local application of 2 × 10^7^ (+4 × 10^7^) stem cells	24 weeks	Reduction in the number of draining fistula—69% of patients Full fistula closure—30% of patients	6 months	20.8%
Dietz et al. [27]	2017	12	Adipose autologous	Local application of 2 × 10^7^ of stem cells on a biological plug	12 weeks 6 months	Complete healing—75% 83% of patients presented with fistula healing	-	N/A
Molendijk et al. + Barnhoorn et al. [19,28]	2015 2020	21	Bone marrow allogenic	Local application of 1-; 3-; 9 × 10^7^ of stem cells	24 weeks	1 × 10^7^ cells—80% 3 × 10^7^ cells—80% 9 × 10^7^ cells—20%	3.5 years	1 × 10^7^ cells—all patients managed to have full fistula closure 3 × 10^7^ cells—0% 9 × 10^7^ cells—0%
Laureti et al. [29]	2020	15	Adipose autologous	Local application of 20 cc of microfragmented adipose tissu stem cells	24 weeks	66.7%	24 weeks	0%
Zhou et al. [30]	2020	22	Adipose autologous	Local application of 5 × 10^6^ stem cells vs. incision-thread-drawing procedure (control group)	1 year	63.6% vs. 54.5% (in control group)	-	N/A

## Data Availability

Not applicable.

## References

[1] Baumgart D.C., Sandborn W.J. (2012). Crohn’s disease. Lancet.

[2] Pogacnik J.S., Salgado G. (2019). Perianal Crohn’s Disease. Clin. Colon Rectal Surg..

[3] Multidisciplinary Team (MDT) Approach to Diagnosis & Management of Perianal Crohn’s Disease | Crohn’s & Colitis Foundation. https://www.crohnscolitisfoundation.org/clinical-pearls/mdt-perianal-crohns-disease.

[4] Panes J., Reinisch W., Rupniewska E., Khan S., Forns J., Khalid J.M., Bojic D., Patel H. (2018). Burden and outcomes for complex perianal fistulas in Crohn’s disease: Systematic review. World J. Gastroenterol..

[5] Herreros M.D., Garcia-Olmo D., Guadalajara H., Georgiev-Hristov T., Brandariz L., Garcia-Arranz M. (2019). Stem cell therapy: A compassionate use program in perianal fistula. Stem Cells Int..

[6] Ding D.C., Shyu W.C., Lin S.Z. (2011). Mesenchymal stem cells. Cell Transplant..

[7] Page M.J., McKenzie J.E., Bossuyt P.M., Boutron I., Hoffmann T.C., Mulrow C.D., Shamseer L., Tetzlaff J.M., Akl E.A., Brennan S.E. (2021). The PRISMA 2020 statement: An updated guideline for reporting systematic reviews. BMJ.

[8] Tozer P.J., Lung P., Lobo A.J., Sebastian S., Brown S.R., Hart A.L., Fearnhead N., Adegbola S.O., Heywood N., Hind D. (2018). Review article: Pathogenesis of Crohn’s perianal fistula—Understanding factors impacting on success and failure of treatment strategies. Aliment. Pharmacol. Ther..

[9] Bernardi L., Dos Santos C.H.M., Pinheiro V.A.Z., Oliveira R.J., Antoniolli-Silva A.C.M.B. (2019). Transplantation of adipose-derived mesenchymal stem cells in refractory crohn’s disease: Systematic review. Arq. Bras. Cir. Dig..

[10] Verstockt B., Ferrante M., Vermeire S., Van Assche G. (2018). New treatment options for inflammatory bowel diseases. J. Gastroenterol..

[11] Hoogduijn M.J., Lombardo E. (2019). Mesenchymal Stromal Cells Anno 2019: Dawn of the Therapeutic Era? Concise Review. Stem Cells Transl. Med..

[12] Carvello M., Lightner A., Yamamoto T., Kotze P.G., Spinelli A. (2019). Mesenchymal Stem Cells for Perianal Crohn’s Disease. Cells.

[13] Georgiev-Hristov T., Guadalajara H., Herreros M.D., Lightner A.L., Dozois E.J., García-Arranz M., García-Olmo D. (2018). A Step-By-Step Surgical Protocol for the Treatment of Perianal Fistula with Adipose-Derived Mesenchymal Stem Cells. J. Gastrointest. Surg..

[14] Banasiewicz T., Eder P., Rydzewska G., Reguła J., Dobrowolska A., Durlik M., Wallner G. (2019). Position of the expert group on the current practice and prospects for the treatment of complex perirectal fistulas in the course of Crohn’s disease. Polish J. Surg..

[15] Herreros M.D., Garcia-Arranz M., Guadalajara H., De-La-Quintana P., Garcia-Olmo D. (2012). Autologous expanded adipose-derived stem cells for the treatment of complex cryptoglandular perianal fistulas: A phase III randomized clinical trial (FATT 1: Fistula advanced therapy trial 1) and long-term evaluation. Dis. Colon Rectum.

[16] Ciccocioppo R., Klersy C., Leffler D.A., Rogers R., Bennett D., Corazza G.R. (2019). Systematic review with meta-analysis: Safety and efficacy of local injections of mesenchymal stem cells in perianal fistulas. JGH Open.

[17] Cao Y., Su Q., Zhang B., Shen F., Li S. (2021). Efficacy of stem cells therapy for Crohn’s fistula: A meta-analysis and systematic review. Stem Cell Res. Ther..

[18] Avivar-Valderas A., Martín-Martín C., Ramírez C., Del Río B., Menta R., Mancheño-Corvo P., Ortiz-Virumbrales M., Herrero-Méndez Á., Panés J., García-Olmo D. (2019). Dissecting allo-sensitization after local administration of human allogeneic adipose mesenchymal stem cells in perianal fistulas of Crohn’s disease patients. Front. Immunol..

[19] Molendijk I., Bonsing B.A., Roelofs H., Peeters K.C.M.J., Wasser M.N.J.M., Dijkstra G., Van Der Woude C.J., Duijvestein M., Veenendaal R.A., Zwaginga J.J. (2015). Allogeneic Bone Marrow-Derived Mesenchymal Stromal Cells Promote Healing of Refractory Perianal Fistulas in Patients With Crohn’s Disease. Gastroenterology.

[20] Panés J., García-Olmo D., Van Assche G., Colombel J.F., Reinisch W., Baumgart D.C., Dignass A., Nachury M., Ferrante M., Kazemi-Shirazi L. (2016). Expanded allogeneic adipose-derived mesenchymal stem cells (Cx601) for complex perianal fistulas in Crohn’s disease: A phase 3 randomised, double-blind controlled trial. Lancet.

[21] Panés J., García-Olmo D., Van Assche G., Colombel J.F., Reinisch W., Baumgart D.C., Dignass A., Nachury M., Ferrante M., Kazemi-Shirazi L. (2018). Long-term Efficacy and Safety of Stem Cell Therapy (Cx601) for Complex Perianal Fistulas in Patients With Crohn’s Disease. Gastroenterology.

[22] Lightner A.L., Ashburn J.H., Brar M.S., Carvello M., Chandrasinghe P., van Overstraeten A.d.B., Fleshner P.R., Gallo G., Kotze P.G., Holubar S.D. (2020). Fistulizing Crohn’s disease. Curr. Probl. Surg..

[23] Garcia-Olmo D., Herreros D., Pascual I., Pascual J.A., Del-Valle E., Zorrilla J., De-La-Quintana P., Garcia-Arranz M., Pascual M. (2009). Expanded Adipose-Derived Stem Cells for the Treatment of Complex Perianal Fistula. Dis. Colon Rectum.

[24] Lee W.Y., Park K.J., Cho Y.B., Yoon S.N., Song K.H., Kim D.S., Jung S.H., Kim M., Yoo H.W., Kim I. (2013). Autologous adipose tissue-derived stem cells treatment demonstrated favorable and sustainable therapeutic effect for crohn’s fistula. Stem Cells.

[25] Cho Y.B., Park K.J., Yoon S.N., Song K.H., Kim D.S., Jung S.H., Kim M., Jeong H.Y., Yu C.S. (2015). Long-Term Results of Adipose-Derived Stem Cell Therapy for the Treatment of Crohn’s Fistula. Stem Cells Transl. Med..

[26] De La Portilla F., Alba F., García-Olmo D., Herrerías J.M., González F.X., Galindo A. (2013). Expanded allogeneic adipose-derived stem cells (eASCs) for the treatment of complex perianal fistula in Crohn’s disease: Results from a multicenter phase I/IIa clinical trial. Int. J. Colorectal Dis..

[27] Dietz A.B., Dozois E.J., Fletcher J.G., Butler G.W., Radel D., Lightner A.L., Dave M., Friton J., Nair A., Camilleri E.T. (2017). Autologous Mesenchymal Stem Cells, Applied in a Bioabsorbable Matrix, for Treatment of Perianal Fistulas in Patients With Crohn’s Disease. Gastroenterology.

[28] Barnhoorn M.C., Wasser M.N.J.M., Roelofs H., Maljaars P.W.J., Molendijk I., Bonsing B.A., Oosten L.E.M., Dijkstra G., Van Der Woude C.J., Roelen D.L. (2020). Long-term evaluation of allogeneic bone marrow-derived mesenchymal stromal cell therapy for Crohn’s disease perianal fistulas. J. Crohn’s Colitis.

[29] Laureti S., Gionchetti P., Cappelli A., Vittori L., Contedini F., Rizzello F., Golfieri R., Campieri M., Poggioli G. (2020). Refractory Complex Crohn’s Perianal Fistulas: A Role for Autologous Microfragmented Adipose Tissue Injection. Inflamm. Bowel Dis..

[30] Zhou C., Li M., Zhang Y., Ni M., Wang Y., Xu D., Shi Y., Zhang B., Chen Y., Huang Y. (2020). Autologous adipose-derived stem cells for the treatment of Crohn’s fistula-in-ano: An open-label, controlled trial. Stem Cell Res. Ther..

[31] Levy O., Kuai R., Siren E.M.J., Bhere D., Milton Y., Nissar N., de Biasio M., Heinelt M., Reeve B., Abdi R. (2020). Shattering barriers toward clinically meaningful MSC therapies. Sci. Adv..

[32] Borycka-Kiciak K., Pietrzak A., Kielar M., Tarnowski W. (2019). Mesenchymal stem cells for the treatment of complex perianal fistulas in patients with Crohn disease. Polish J. Surg..

[33] Lopez N., Ramamoorthy S., Sandborn W.J. (2019). Recent advances in the management of perianal fistulizing Crohn’s disease: Lessons for the clinic. Expert Rev. Gastroenterol. Hepatol..

[34] Gallo G., Tiesi V., Fulginiti S., De Paola G., Vescio G., Sammarco G. (2020). Mesenchymal Stromal Cell Therapy in the Management of Perianal Fistulas in Crohn’s Disease: An Up-To-Date Review. Medicina.

[35] Lightner A.L., Wang Z., Zubair A.C., Dozois E.J. (2018). A systematic review and meta-analysis of mesenchymal stem cell injections for the treatment of perianal Crohn’s disease: Progress made and future directions. Dis. Colon Rectum.

